# Primary and Secondary Spontaneous Pneumothorax: Prevalence, Clinical Features, and In-Hospital Mortality

**DOI:** 10.1155/2017/6014967

**Published:** 2017-03-13

**Authors:** Takuya Onuki, Sho Ueda, Masatoshi Yamaoka, Yoshiaki Sekiya, Hitoshi Yamada, Naoki Kawakami, Yuichi Araki, Yoko Wakai, Kazuhito Saito, Masaharu Inagaki, Naoki Matsumiya

**Affiliations:** ^1^Department of General Thoracic Surgery, Tsuchiura Kyodo General Hospital, 4-1-1 Otsuno, Tsuchiura, Ibaraki 300-0028, Japan; ^2^Department of Emergency Medicine, Tsuchiura Kyodo General Hospital, 4-1-1 Otsuno, Tsuchiura, Ibaraki 300-0028, Japan; ^3^Department of Respiratory Medicine, Tsuchiura Kyodo General Hospital, 4-1-1 Otsuno, Tsuchiura, Ibaraki 300-0028, Japan

## Abstract

*Background*. Optimal treatment practices and factors associated with in-hospital mortality in spontaneous pneumothorax (SP) are not fully understood. We evaluated prevalence, clinical characteristics, and in-hospital mortality among Japanese patients with primary or secondary SP (PSP/SSP).* Methods*. We retrospectively reviewed and stratified 938 instances of pneumothorax in 751 consecutive patients diagnosed with SP into the PSP and SSP groups. Factors associated with in-hospital mortality in SSP were identified by multiple logistic regression analysis.* Results*. In the SSP group (*n* = 327; 34.9%), patient age, requirement for emergency transport, and length of stay were greater (all, *p* < 0.001), while the prevalence of smoking (*p* = 0.023) and number of surgical interventions (*p* < 0.001) were lower compared to those in the PSP group (*n* = 611; 65.1%). Among the 16 in-hospital deceased patients, 12 (75.0%) received emergency transportation and 10 (62.5%) exhibited performance status (PS) of 3-4. In the SSP group, emergency transportation was an independent factor for in-hospital mortality (odds ratio 16.37; 95% confidence interval, 4.85–55.20; *p* < 0.001).* Conclusions*. The prevalence and clinical characteristics of PSP and SSP differ considerably. Patients with SSP receiving emergency transportation should receive careful attention.

## 1. Introduction

Pneumothorax is a thoracic disorder manifested as abnormal collection of air in the pleural space [1, 2]. Pneumothorax can be caused by blunt or penetrating chest injuries, medical procedures, or damage from underlying lung diseases [3–5]. Spontaneous pneumothorax (SP) is a type of pneumothorax that develops in the absence of trauma [3–6]. It is further classified as primary and secondary SP (PSP/SSP). While PSP affects patients with no clinically apparent lung disorders, SSP involves an underlying pulmonary disease, which most often is chronic obstructive pulmonary disease (COPD) [3, 7]. Spontaneous pneumothorax is a significant health burden, with annual incidences of 18–28 and 1.2–6 cases per 100,000 men and women, respectively [4, 5, 8]. The annual incidences of PSP among men and women are 7.4–18 (age-adjusted incidence) and 1.2–6 cases per 100,000 population, respectively; the annual incidences of SSP are similar, approximately 6.3 and 2 cases per 100,000 men and women, respectively [8–11].

Risk factors for PSP include tall-and-thin body shape, maleness, and smoking [12]. In contrast, a multitude of respiratory disorders have been described as causes of SSP [13]. The most frequent underlying disorders in SSP are COPD with emphysema, cystic fibrosis, tuberculosis, lung cancer, interstitial pneumonitis, and human immunodeficiency virus-associated* Pneumocystis carinii *pneumonia [6, 14–16]. While PSP typically occurs between the ages of 10 and 30 years, the peak incidence of SSP is observed in later years—between the ages of 60 and 64 years—depending on the underlying condition [4]. Most importantly, lung function in patients with SSP is already compromised; therefore, unlike PSP, SSP often presents as a potentially life-threatening disease, requiring immediate action [10, 17–19]. Though guidelines for management of pneumothorax are available, considerable variations in clinical practice have been reported in studies carried out in various countries [20–22]. Furthermore, most studies have only described the practices for management of PSP, and not much emphasis has been placed on the management of SSP or understanding of the clinical and demographic differences between PSP and SSP. Early initiation of treatment is expected to be a determinant of successful outcome in SSP [23]. Furthermore, little is understood regarding the roles of risk factors of in-hospital mortality in SP, and only a few studies have investigated the demographic and clinical features of Japanese patients with SP/PSP/SSP [24–28]. However, the heterogeneity of symptoms of SP and geographical variations in clinical practice demand a careful understanding of clinical presentation, management practices, and treatment outcomes of the disease [17].

This study analyzed the differences in demographic and clinical features between patients with PSP and SSP in a Japanese population. Since early treatment and prompt clinical assessment are the cornerstones of successful clinical outcome, we hypothesized that “emergency transportation”, that is, transport of a patient with SP by ambulance to the emergency department (ED), might be a critical factor in the management of patients with SSP. To this end, factors associated with in-hospital mortality were identified by multivariate logistic regression analysis. Resolution of pneumothorax, treatment protocol, and underlying diseases were also analyzed in case of patients who died during hospitalization.

## 2. Materials and Methods

### 2.1. Study Design and Exclusion Criteria

This retrospective study included patients who presented with the diagnosis of “pneumothorax” at the Tsuchiura Kyodo General Hospital, Ibaraki, Japan, between January 2004 and December 2014. Patient records were retrieved by the electronic clinical data analysis and retrieval system according to the International Classification of Diseases, Ninth Revision, codes 512.0 (spontaneous pneumothorax). Patients with traumatic, iatrogenic, or wrong diagnoses as well as those below the age of 10 years were excluded. Patients who were initially transferred from another hospital for management were included but not considered under the category of “emergency transportation” (defined later). In this study, SPs that did not result in hospitalization were not included. The number of pneumothoraxes was determined, and multiple entries were considered to include metachronous pneumothoraxes. The institutional review board of the Tsuchiura Kyodo General Hospital approved this study (Approval Number: 533) and written oral informed consent was received from majority of the participants while the rest of them provided only verbal consent.

### 2.2. Evaluation

Data including demographic information, type of pneumothorax, laterality, smoking status, emergency transportation, surgical intervention, in-hospital mortality, and length of stay (LOS) were collected. X-ray radiographs of all included patients were retrieved. Underlying pulmonary diseases were recorded for all patients with SSP.

### 2.3. Definition and Stratification

Primary SP was defined as SP in a person without an underlying lung disease, whereas SSP was defined as SP with an underlying lung disease [1, 2]. Laterality was categorized as right, left, simultaneous bilateral, or unknown. Past and active smokers were assigned to the smokers' group. Underlying pulmonary diseases in patients with SSP were surveyed, and multiple diseases were included. Symptom onset was defined as onset of symptoms of pleuritic chest pain or dyspnea. Emergency transportation was defined for patients who phoned in for an ambulance and were transported by ambulance to our ED. Patients transferred to our hospital by ambulance from another clinical institute were not assigned to the “emergency transportation” category. Patients transported to the ED were preferentially examined by the doctor over other patients, and exclusive treatment was started promptly. The duration of a single episode of hospitalization, defined as the LOS, was calculated from the day of admission to that of discharge. In-hospital mortality was defined as death from any cause during hospitalization. Patients who received emergency transportation were also evaluated for performance status (PS), which was determined according to the criteria published by the Eastern Cooperative Oncology Group and modified for this study (Table 1) [29]. Factors found to be significant indicators of mortality were further evaluated by multivariate analysis to identify independent prognosticators of in-hospital mortality in patients with SSP. Cure was defined by the complete stoppage of air leakage and absence of recurrence of SP.

### 2.4. Treatment Modalities

Treatment modalities included oxygen inhalation (OI), chest drainage with a chest tube (16–24 Fr), and video-assisted thoracoscopic surgery (VATS) with wedge resection, which was performed in case of recurrence or failure of treatment by OI and simple chest tube insertion. Mild SP was treated by observation only or by continuous chest drainage for a few days. Recurrence rate was defined by the percentage of patients who exhibited SP during the follow-up period. Patients who received nonsurgical treatment were followed-up for a few weeks, while those who underwent surgery were followed up for 2-3 months. Patients exhibiting prolonged air leakage and recurrence of SP were treated by surgery, while those suspected as being unfit for surgical intervention were treated by pleurodesis with tetracycline, OK-432, and talc.

### 2.5. Statistical Analysis

Baseline characteristics were described using descriptive statistics. Patient characteristics and treatment outcomes were compared by the chi-square test, Fisher's exact test, or *t*-test, as appropriate. Categorical variables were represented as frequencies and percentages. Continuous variables with standard distribution were represented as mean values and standard derivations. Predictors of in-hospital mortality in SSP were identified by multivariate logistic regression analysis. Evaluated parameters included sex, laterality, smoking status, and emergency transportation. The SPSS version 22 software (IBM Japan, Tokyo, Japan) was used for statistical analysis. Differences were considered significant at values of *p* < 0.05.

## 3. Results

### 3.1. Patients

Of the 751 patients (male, 649; 86.4%) included in the present study, 142 (18.9%) presented with metachronous SP. While 485 (64.5%) patients exhibited PSP, 266 (35.4%) presented with SSP. There were no differences in sex between the PSP and SSP groups (proportion of male patients: PSP versus SSP, 86.6% versus 86.1%; *p* = 0.846).

### 3.2. Pneumothoraxes, Treatment Methods, and Recurrence Rates

The total number of pneumothoraxes among the included patients was 938, with 13.8% of pneumothoraxes being observed in women. The mean age at the time of presentation of pneumothorax was 43 ± 13 years. While 48% of pneumothoraxes were left lateral, only 1.2% were bilateral. A significant proportion of patients exhibiting pneumothoraxes were smokers (65.7%), while 50.8% received surgical treatment, and 8.7% received emergency transportation. In-hospital mortality was 1.7%, and the average LOS was 11 ± 13 days (Table 2). The underlying reasons for emergency transport (*n* = 82) were complaints of dyspnea (*n* = 51; 62.2%), dyspnea and chest pain (*n* = 8; 9.8%), disturbance of consciousness (*n* = 7; 8.5%), chest pain (*n* = 6; 7.3%), dyspnea and disturbance of consciousness (*n* = 2; 2.4%), and others (*n* = 8; 9.8%).

With regard to treatment modality, 459 (48.9%) patients received chest drainage; 54 (5.8%) patients received surgery; 423 (45.1%) patients received surgery after chest drainage; and 2 patients (0.2%) only required observation (Table 3). In the PSP and SSP groups, the recurrence rates among patients who received nonsurgical treatment were 24.5% and 17.4%, while those among patients who underwent surgery were 2.7% and 1.9%, respectively (Table 4).

### 3.3. PSP and SSP

In the present study, 65.1% of the pneumothoraxes were PSP. The mean age at the time of presentation of pneumothorax in the PSP group was significantly lower compared to that in the SSP group (27 ± 12 years versus 70 ± 14 years; *p* < 0.001). The prevalence of pneumothorax among men in the PSP and SSP groups was 65.9% and 34.1%, respectively. There was no significant difference in the distribution of men or women with SP between the two groups (*p* = 0.695). The proportions of smokers in the PSP and SSP groups were 67.8% and 32.3%, respectively (*p* = 0.023). The proportions of left, right, and bilateral SP in the PSP group were 51.4%, 47.2%, and 1.3%, respectively, while the corresponding proportions in the SSP group were 41.6%, 57.5%, and 0.9%, respectively. Emergency transportation was required by 2.4% and 20.5% of patients in the PSP and SSP groups, respectively (*p* < 0.001). The SSP group included 4 patients with PS of 4; even upon exclusion of these 4 patients from analysis, the proportion of patients requiring emergency transportation in the SSP group was higher compared to that in the PSP group (*p* < 0.05). The number of patients requiring surgical intervention in the PSP group was higher compared to that in the SSP group (61.2% versus 31.5%; *p* < 0.001). The LOS and in-hospital mortality among patients in the SSP group were greater compared to those in the PSP group (both, *p* < 0.001). Only 2 of 16 patients (12.5%) who died during hospitalization received surgical intervention, and both had presented with SSP. In-hospital mortality among all patients with SSP and among patients with SSP who underwent surgery was 4.6% and 1.9%, respectively (*p* = 0.0934).

### 3.4. Underlying Pulmonary Diseases

The underlying pulmonary diseases in 266 patients of the SSP group are summarized in Table 5. The most frequent diseases were pulmonary emphysema, that is, COPD, (73.3%), interstitial pneumonitis including pulmonary fibrosis (7.9%), and lung cancer (7.5%).

### 3.5. Risk Factors for In-Hospital Mortality in SSP

In the SSP group, multivariate analysis of sex, laterality, smoking status, and emergency transportation revealed only emergency transportation (OR, 16.47; 95% CI, 4.85–55.20; *p* < 0.001) as independent risk factors for in-hospital mortality in patients with SSP (Table 6).

### 3.6. Clinical Characteristics of the Deceased Patients

Tables 7 and 8 present the results of analysis of therapeutic approaches, LOS, treatment outcomes, and main causes of death among patients with SP who died during hospitalization. Among the 16 cases of in-hospital mortality, 14 (87.5%) patients died of respiratory diseases, and 6 (37.5%) died after resolution of SP. The mean age of these patients was 80 ± 9 years, and the mean LOS was 41 ± 65 days. While 12 (75.0%) of these patients had received emergency transportation, 10 (62.5%) had exhibited PS of 3 or 4, and 15 (93.8%) had presented with SSP. While 12 (75.0%) patients had received continuous chest drainage, only 2 (16.7%) had received surgical intervention. Although 37.5% of patients who died during hospitalization exhibited PS ≤ 2, PS 4 had a significant effect on in-hospital mortality (PS = 4 versus PS < 4, 75% versus 1.7%; *p* < 0.0001).

## 4. Discussion

This study reports that emergency transportation is an independent factor associated with in-hospital mortality in SSP. Our results further suggest that PSP and SSP have several significantly different clinical and demographic features. Old age, right laterality, and nonsmoking status were more prevalent in the SSP group than in the PSP group, while surgical interventions and left laterality were more frequent in latter. There were more men than women in both groups. The proportion of women with SSP was greater compared to that with PSP, although the difference did not reach statistical significance.

The most frequent underlying pulmonary disease in SSP was pulmonary emphysema, which corresponds with previous reports [15, 30]. The second most frequent disease was interstitial pneumonitis or pulmonary fibrosis; this result is consistent with that reported by Ichinose et al., who described surgical interventions employed in Japanese patients with SSP [15]. Interestingly, Brown et al. reported bronchial asthma as the most frequent underlying pulmonary disease among Australian patients with SP [30]. It should be noted that the overall prevalence of underlying diseases might vary between Western and Asian populations depending on ethnic, social, and other factors. Such differences might also affect the prevalence of SSP and its clinical features, thus explaining the discrepancies between the results of the present study and those of Brown et al. However, the present results suggesting that right laterality is more common in SSP than in PSP are in agreement with those of Brown et al. [30]. This result can be explained by the fact that pulmonary emphysema-associated azygoesophageal recess is more common on the right side than on the left, and since pulmonary emphysema is one of the more common underlying diseases in SSP, right laterality is expected to be relatively more prevalent in SSP than in PSP [31].

There were significant differences in emergency transportation, surgical intervention, LOS, and in-hospital mortality between the PSP and SSP groups in the present study. The requirement for emergency transportation, LOS, and in-hospital mortality in the SSP group was much greater compared to those in the PSP group. However, the SSP group was not homogeneous in composition. It comprised an older population than the PSP group, and the clinical manifestations of SSP varied among the patients, depending on the underlying pulmonary disease. Therefore, the SSP group tended to involve more instances of emergency transportation and longer hospital stay than the PSP group. However, in multivariate analysis involving sex, laterality, smoking status, and emergency transportation as variables, only emergency transportation was found to be independent factors associated with in-hospital mortality.

The frequency of surgical interventions in the SSP group was considerably lower compared to that in the PSP group, which might seem contradictory to the results of recent studies that established the efficacy of surgical intervention for SSP and advocated in favor of video-assisted thoracic surgery [15, 16]. However, it is suspected that many patients with SSP are denied surgery because of their inability to endure the procedure; therefore, our results do not necessarily reflect the low clinical efficacy of surgical intervention. It must be mentioned here that the in-hospital mortality rate among all patients with SSP and among patients with SSP who underwent surgery was 4.6% and 1.9%, respectively; these proportions were comparable with those reported previously (0–6.0%) [15, 16, 19].

Among patients who received emergency transportation, only 3 presented with light pneumothorax and, therefore, did not receive invasive treatment. All other patients (79 cases of SP) received chest drainage in the ED as soon as possible after diagnosis of pneumothorax by X-ray radiography. Since only patients with PS 4 have to rely on emergency transportation, we performed subgroup analysis of the data after excluding patients exhibiting PS 4. Despite this exclusion, emergency transportation remained an independent factor for in-hospital mortality. This suggests that, at least among Japanese patients, clinicians should more carefully treat patients with SSP who are being transported in emergency. It may be noted that, among the 16 patients who died during hospitalization, 12 (75.0%) had received emergency transportation, 15 (93.8%) had presented with SSP, and 10 (62.5%) had exhibited PS of 3 or 4.

There are some limitations to this study. The study was of retrospective, observational, and single-institution design. Although there were no major changes in therapeutic strategies for SP during the study period, minor changes, such as new antibiotics or surgical devices, might have been implemented. Therefore, small therapeutic changes could have existed between the early and late periods of the study. Since emergency transportation systems vary among countries, it is necessary to consider country-specific factors while generalizing our results. All of the patients who died during hospitalization were above 60 years of age and had underlying diseases, which precluded confounding control for age and/or comorbidity. Further studies are required to analyze the role of these potential confounders. Because it was not possible to evaluate the PS in all 938 instances of pneumothorax, we could not include PS as a confounder in multivariate analysis. However, 37.5% of patients who died during hospitalization exhibited PS ≤ 2, and our results revealed a significantly higher rate of in-hospital mortality among patients with PS = 4 than among patients with PS < 4, which suggests that size and severity of pneumothorax, age, performance status, body mass index, and SP-associated comorbidities should to be analyzed in future studies to fully establish “emergency transportation” as an independent risk factor. We stress that our results should be interpreted within these limitations. Nevertheless, the present results are noteworthy for highlighting the possible role of emergency transportation in in-hospital mortality among patients with SP and presenting the associated clinical features of SP in the Japanese population. This study is expected to stimulate further research on identifying factors that might allow better clinical management of such high-risk patients.

## 5. Conclusions

Sex, age, laterality, and smoking status vary significantly between patients with PSP and SSP in Japan. Our results also underscore the significance of emergency transportation for patients with SSP and suggest that patients with SSP receiving emergency transportation should receive careful attention irrespective of their PS. To the best of our knowledge, this is the first report on the significance of emergency transportation for patients with PSP and SSP in the Japanese population.

## Figures and Tables

**Table 1:** Modified ECOG performance status for this study.

Grade	Content
0	Fully active without restriction
1	Restricted in physically strenuous activities, but ambulatory and able to carry out work of a light or sedentary nature
2	Ambulatory and capable of all self-care, but unable to carry out any work activities; up and about for more than 50% of waking hours
3	Capable of only limited self-care; confined to bed or a chair for more than 50% of waking hours
4	Completely disabled; cannot perform any self-care; completely confined to bed or a chair

ECOG, Eastern Cooperative Oncology Group.

**Table 2:** Demographic distribution and clinical features of spontaneous pneumothorax.

	SP	PSP	SSP	*p* value
Number of pneumothoraxes	938	611 (65.1)	327 (34.9)	
Patients	751	485 (64.6)	266 (35.4)	
Age at the time of pneumothorax (Mean ± SD)	43 ± 13	27 ± 12	70 ± 14	<0.001^*∗*^
Sex-wise distribution of pneumothoraxes				
Men	809 (86.2)	533 (65.9)	276 (34.1)	0.695^*∗*^
Women	129 (13.8)	78 (60.5)	51 (39.5)	
Laterality				
Left	450 (48.0)	314 (51.4)	136 (41.6)	0.022^#^
Right	476 (50.7)	288 (47.2)	188 (57.5)	
Bilateral	11 (1.2)	8 (1.3)	3 (0.9)	
Unknown	1 (0.1)	1 (0.1)	0 (0.0)	
Smoking status				
Smoker	593 (63.2)	402 (67.8)	191 (32.2)	0.023^$^
Never	336 (35.7)	203 (60.4)	133 (39.6)	
Unknown	9 (0.1)	6 (66.7)	3 (33.3)	
Emergency transportation (%)	82 (8.7)	15 (2.4)	67 (20.5)	<0.001^*∗*^
Surgical intervention (%)	477 (50.8)	374 (61.2)	103 (31.5)	<0.001^*∗*^
LOS days (median ± SD)	11 ± 13 (8)	9 ± 5 (8)	16 ± 20 (11)	<0.001^*∗*^
In-hospital mortality	16 (1.71)	1 (0.16)	15 (4.59)	<0.001^*∗*^

Data are presented as *n* (%) unless otherwise specified. ^*∗*^Comparison between the SSP and PSP groups; ^#^comparison of pneumothoraxes of left, right, bi, and unknown laterality among patients with PSP and SSP (4 × 2 contingency table); ^$^comparison of smokers among patients with PSP and SSP. SP, spontaneous pneumothorax; PSP, primary SP; SSP, secondary SP; SD, standard deviation; LOS, length of stay.

**Table 3:** Therapeutic methods employed for management of spontaneous pneumothorax.

Chest drainage (with/without pleurodesis)	459
Surgery (with/without pleurodesis)	54
Chest drainage followed by surgery (with/without pleurodesis)	423
Observation only	2

**Table 4:** Recurrence rates of pneumothorax in the PSP and SSP groups.

	Cases	(%)
Nonsurgical treatment (drainage with/without pleurodesis or observation only)		
PSP	58	24.5
SSP	39	17.4
Surgery (with/without pleurodesis)		
PSP	10	2.7
SSP	2	1.9

PSP, primary spontaneous pneumothorax; SSP, secondary spontaneous pneumothorax.

**Table 5:** Underlying pulmonary diseases in patients with secondary spontaneous pneumothorax (*N* = 266).

Disease	*n* (%)
Pulmonary emphysema	195 (73.3)
Interstitial pneumonitis or pulmonary fibrosis	21 (7.9)
Lung cancer	20 (7.5)
Infectious disease (pneumonia, pneumomycosis, etc.)	12 (4.5)
Catamenial pneumothorax	8 (3.0)
Nontuberculous mycobacterial infection	7 (2.6)
Birt-Hogg-Dubé syndrome	4 (1.5)
Obsolete pulmonary tuberculosis	3 (1.2)
Others	9 (3.4)

**Table 6:** Multivariate analysis of factors associated with in-hospital mortality in patients with secondary spontaneous pneumothorax.

	OR	95% CI	*p* value
Sex	3.9	0.94–16.25	0.06
Laterality	0.59	0.19–1.78	0.35
Smoking status	1.17	0.30–4.49	0.82
Emergency transportation	16.37	4.85–55.20	<0.001

OR, odds ratio; CI, confidence interval.

**Table 7:** Demographic and clinical details of patients with spontaneous pneumothorax according to requirement for emergency transportation.

Patients	Age (years)	Sex	Laterality	Emergency transportation	Smoking status	PS before pneumothorax	PSP/SSP	Existing respiratory diseases
1	62	M	R	Yes	Yes	4	SSP	Lung cancer
2	68	M	L	Yes	Yes	2	SSP	Pulmonary emphysema, pneumonia
3	72	M	R	No	Yes	1	SSP	Pulmonary emphysema
4	72	M	R	Yes	Yes	2	SSP	Pulmonary emphysema
5	74	M	R	Yes	Unknown	3	SSP	Pneumonia
6	76	M	R	Yes	Yes	0	SSP	Lung cancer, pneumonia
7	77	M	L	Yes	Yes	1	SSP	Pulmonary emphysema
8	78	M	L	No	Never	3	SSP	Pulmonary emphysema
9	79	F	R	No	Yes	2	SSP	Pneumonia, pyothorax
10	80	M	L	Yes	Yes	3	SSP	Pulmonary emphysema, pneumomycosis
11	85	F	L	Yes	Never	4	SSP	Interstitial pneumonitis
12	86	M	R	Yes	Yes	3	SSP	Interstitial pneumonitis
13	87	F	R	Yes	Never	4	SSP	Lung cancer
14	87	M	L	Yes	Yes	3	SSP	Pulmonary emphysema, pneumonia
15	94	F	R	No	Never	3	SSP	Obsolete tuberculosis
16	96	M	R	Yes	Yes	3	PSP	—

L, left; R, right; PS, performance status according to the Eastern Cooperative Oncology Group; PSP, primary spontaneous pneumothorax; SSP, secondary spontaneous pneumothorax.

**Table 8:** Therapy, LOS, progress of pneumothorax, and cause of death in patients with spontaneous pneumothorax.

Patients	Therapy	LOS (days)	Progress of pneumothorax	Direct cause of death
1	CD + pleurodesis	14	NC	Lung cancer
2	CD + pleurodesis	16	Cured	Pneumonia
3	Surgery	13	NC	Pyothorax, postoperative respiratory failure
4	Surgery	274	Cured	Post-operative respiratory failure
5	CD	5	NC	Pneumonia
6	CD	56	Cure	Lung cancer
7	CD	8	Cure	Exacerbation of chronic respiratory failure
8	CD + pleurodesis	15	Cure	Pyothorax
9	CD + pleurodesis	23	NC	Pneumothorax followed by respiratory failure
10	CD + pleurodesis	23	NC	Sepsis followed by multiple organ failure
11	Observation only	30	NC	Pneumothorax followed by respiratory failure
12	CD	19	NC	Intestinal pneumonitis
13	Observation only	66	NC	Pneumothorax followed by general prostration
14	CD	8	NC	Cardiac disease (details unknown)
15	CD	32	NC	Pyothorax, respiratory failure
16	CD + pleurodesis	59	Cure	Pneumothorax followed by multiple organ failure

CD, chest drainage; LOS, length of stay; NC, no change.
